# The State of the Art on PVDF Membrane Preparation for Membrane Distillation and Membrane Crystallization: Towards the Use of Non-Toxic Solvents

**DOI:** 10.3390/membranes15040117

**Published:** 2025-04-08

**Authors:** Aqsa Mansoor Khan, Francesca Russo, Francesca Macedonio, Alessandra Criscuoli, Efrem Curcio, Alberto Figoli

**Affiliations:** 1 Institute for Membrane Technology, National Research Council Italy CNR-ITM, Via P.Bucci 17/C, 87036 Rende, CS, Italy; aqsamansoor.khan@unical.it (A.M.K.); f.russo@itm.cnr.it (F.R.); a.figoli@itm.cnr.it (A.F.); 2 Department of Environmental Engineering, DIAM, University of Calabria, Via P.Bucci-Cube 44/A, 87036 Rende, CS, Italy; efrem.curcio@unical.it

**Keywords:** PVDF membranes, green solvents, membrane distillation, membrane crystallization

## Abstract

Most parts of the earth are covered with water, but only 0.3% of it is available to living beings. Industrial growth, fast urbanization, and poor water management have badly affected the water quality. In recent years, a transition has been seen from the traditional (physical, chemical) wastewater treatment methods towards a greener, sustainable, and scalable membrane technology. Even though membrane technology offers a green pathway to address the wastewater treatment issue on a larger scale, the fabrication of polymeric membranes from toxic solvents is an obstacle in making it a fully green method. The concept of green chemistry has encouraged scientists to engage in research for new biodegradable and non-protic solvents to replace with already existing toxic ones. This review outlines the use of non-toxic solvents for the preparation of PVDF membranes and their application in membrane distillation and membrane crystallization.

## 1. Introduction

Water is the basic commodity required for life functioning. Uneven population spikes, increased living standards, and climatic changes are some of the grounds that caused the dwindling of water reserves with a much higher rate. Alternatives to fresh water sources, the use of unconventional water systems such as industrial wastewater, brackish sea water, and industrial brine, etc., have gained the interest of scientists [1,2].

Scientists have furnished suitable water quality to be used at industrial level in irrigation of crops. Several treatment approaches have been employed over the last two decades to treat water for reuse. Chemical precipitation [3], coagulation [4], and flocculation [5], flotation [6], filtration [7], adsorption [8], ion exchange [9], evaporation [10], desalination [11], and other biological treatment procedures are widely used in wastewater treatment and desalination. The majority of these procedures, like flocculation and coagulation, produce sludge, which adds an additional challenge to sludge disposal since it results in secondary contaminant. Few treatment procedures including evaporation, liquid–liquid extraction, flotation, froth flotation, etc., demand a significant amount of energy for the remediation of water, which makes it an expensive procedure [2]. With mounting concerns about non-renewable resource depletion and environmental challenges, meeting future energy demands in a sustainable manner is a pressing need. Therefore, the use of water recycling and desalination plants has appeared as a crucial method for sustaining next generations. The current global desalination contracted capacity is around 109.22 million m^3^/d [12]. Limiting aspects associated with it are the pre-treatment, economic factor, and environmental footprint of dumping the concentrated effluents into ocean and often on the earth’s surface [13]. Considerable effort has been put forth in green technology development for clean water supply [14]. Over the years, membrane technology has become a leading and emerging separation technology [15,16]. It covers major industrial applications like the treatment of toxic industrial waste, wastewater coming from steel, oil, and chemical industries, power production units, membranes that also serve in energy storage and conversion, gas and vapor separation, food processing, the desalination of brackish and sea water, pollution control (air), in medical fields like hemodialysis, the separation of proteins and micro-organisms, etc. [17]. Membrane engineering offers a green pathway for freshwater production. Over the period, it achieves boost in wastewater treatment and also fulfills the demand for process intensification, by offering advantages in size reduction in the plant, economic factors, and low-energy operations. Membrane technology continues to expand in the advancement of new and novel membrane material for better mechanical and thermal properties as well as improved permeability and selectivity properties [18]. The design aims to focus on developing methodologies that in turn enhance the plant efficiency, reduce the energy consumption, cut capital expenditures, and lower the environmental impact.

Membrane distillation is an advancing thermally driven separation technique that uses the porous hydrophobic membrane layer, which hinders the direct permeation of feed water, and it separates the feed (dissolved components) with the vapor phase. Liquids in the form of vapors are allowed to pass through the membranes, essential for contamination rejection. In comparison with other separation techniques, the reverse osmosis (RO) pore size of membranes used in MD is large, which reduces the chances of membrane fouling [19,20]. Unlike classical pressure-driven filtration processes such as reverse osmosis (RO) [21], where high transmembrane pressure forces water through the membrane and leaves behind solute particles, thereby leading to concentration polarization and fouling, the liquid streams in MD are recirculated at nearly atmospheric pressure. This low-pressure gradient reduces the forces that compact fouling onto the membrane surface. Although concentration and temperature polarization can occur in MD due to its thermally driven nature, the recirculation and induced turbulence effectively mix the solute layer near the membrane surface. This dynamic mixing minimizes the formation of the compact fouling layer, thereby enhancing membrane performance even with high saline feed water [22]. MD can be utilized to remove low-boiling point organic components and to recover purified water from wastewater streams and aqueous solutions [23]. It can also be used to produce oversaturated solutions for crystallization purposes (membrane crystallization).

This paper provides the state of PVDF membranes in membrane distillation and membrane crystallization. This study begins with an introduction of membrane technology in desalination, particularly focusing on membrane distillation, highlights its principles and the different configurations and types of membrane geometries used. It explains membrane characteristics for membrane distillation, the fabrication of PVDF membranes via phase separation techniques using toxic solvents and transitions to their alternative bio-degradable solvents for sustainable membrane fabrication. The performance of PVDF membranes in terms of flux and rejection are evaluated using sodium chloride (NaCl) salt solution. This review also provides an overview of membrane crystallization, its principles, configurations, and membranes used in MCr. The estimation of the flux with a high concentration of salt solution with membranes prepared with toxic/non-toxic solvent is reported. Future perspectives of the role of green solvents in the circular economy and challenges associated with the scale-up of the membrane production are discussed.

## 2. Membrane Technology in Desalination

Membrane technology has emerged as promising technique, in various fields like food industry, biotechnology, desalination and water treatment processes, etc., owing to its easy scale-up, low economic cost, and benign operating conditions with high efficiency. It covers various separation techniques for desalination like, electrodialysis (ED) [24], nanofiltration (NF) [25], reverse osmosis (RO) [26], and membrane distillation (MD) [27]. Electrodialysis presents a high recovery rate of 94%, and a longer membrane life of 15 years can be achieved with a 98% rate of recovery if coupled with reverse osmosis (RO) and operated appropriately.

However, capital costs associated with this are still very high [28]. Pressure-driven membrane technologies such as RO and NF are often being used at the commercial scale to have clean water [29]. To overcome freshwater deficit, reverse osmosis plays an important role in desalination of the sea water with its low rate of energy consumption, and it produces more than 70% of the total clean water world-wide [30]. Guizi Chen et al. [31] reported some major limitations associated with the RO plant that are membrane fouling, the low amount of recovered water due to osmotic-pressure, and the production of high concentrate. Disposal management of the concentrated brine is a major issue, and it leads to secondary pollution due to the discharge of the brine. In comparison to the reverse osmosis system, membrane distillation, which is not fully commercialized yet, exhibits more than a 99.9% solute rejection rate and is less affected by osmotic limitations [32]. For the past few years, membrane-based technologies have taken over the conventional temperature-driven distillation columns, owing to their energy friendly properties.

## 3. Membrane Distillation

### 3.1. Principles of Membrane Distillation

**Figure 1:**
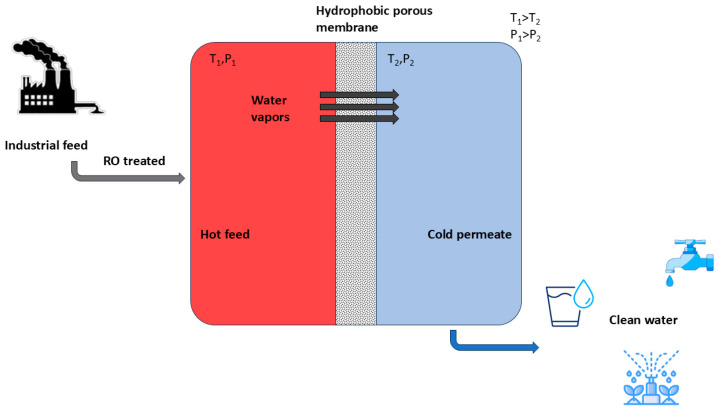
Schematic representation of MD for the recovery of fresh water from industrial feed. Reprinted from [36] with open access.

Membrane distillation (MD) is a thermally driven separation process that uses trans-membrane vapor pressure as a driving force. MD provides even higher water quality (i.e., 100% theoretical rejection of ions) than reverse osmosis (RO). It is a non-isothermal membrane-based separation technique that uses a hydrophobic porous layer as a separating medium between two compartments. Figure 1 illustrates the operation of membrane distillation setup (e.g., in direct contact, MD streams at different temperature flow in the two compartments: hot feed (saline water) and the cold permeate (distillate water)). The feed must come in contact with the hydrophobic surface of the membrane. The temperature of feed side can be kept as low as 40 °C [33]. The hydrophobic layer only allows the passage of water in the form of vapors on the permeate side of the membrane, retaining the non-volatile components on the feed side of the membrane. The features that make membrane distillation of interest compared to other thermal desalinating methods that include multistage flash distillation (MSF), multi-effect distillation (MED), and vapor compression [34] are the following: (1) the low operating temperature conditions because feed (water containing salts) is not necessarily heated up to the boiling point; (2) the possibility to treat high-concentrated feeds; and (3) the higher compactness. The low operating temperatures allow the use of alternative low-energy sources like geothermal, sunlight, and heat generated from industrial waste, which can lead to low carbon footprints. Moreover, membranes used in the MD system are highly porous with larger pore size as compared to reverse osmosis (RO) [33]; this makes them less prone to fouling and reduces the mass transfer resistance and the heat conduction through membrane. The high packing density of the membrane modules and porosity of the membranes increases the area of specific evaporation, which in turns increases the rate of clean water production by reducing the surface area needed for the desalination plant, with respect to conventional distillation columns [35].

The performance of the membranes is evaluated mainly by two parameters, i.e., the transmembrane flux (J) and rejection rate of species (R). These are calculated using the following equations:

(1) $$\mathrm{Flux}=J=\frac{Quantity\;of\;Permeate\;(kg)}{Membrane\;area\;\left(A\right)\times Time\;required\;to\;collect\;premeate\;(h)}$$

(2) $$\mathrm{Rejection}=R=\frac{C_{f}-C_{p}}{C_{f}}=1-\frac{C_{p}}{C_{f}}$$

where C_f_ is feed concentration, and C_p_ is the concentration of permeate.

**Figure 2:**
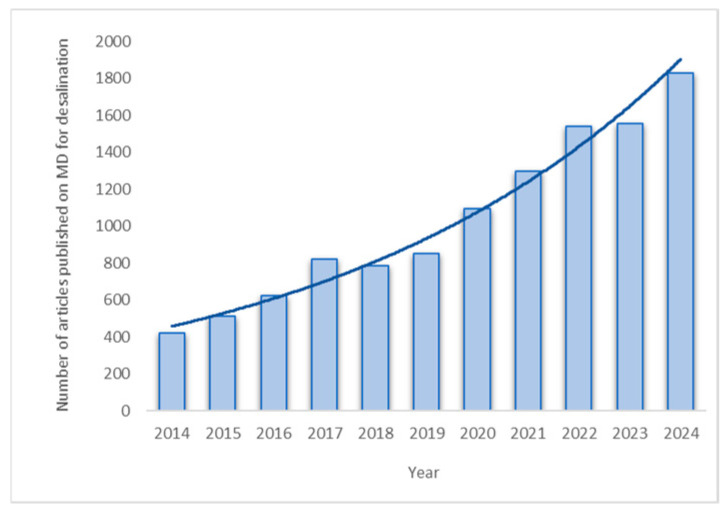
Number of published articles on membrane distillation for desalination in last 10 years (updated until 20 January 2025). Gateway: Science Direct using keywords membrane distillation and desalination.

Over the last decade, an increase has been seen in the research area for potable water by membrane distillation. In particular, Figure 2 represents the number of published articles on membrane distillation for desalination.

### 3.2. Configurations of MD

**Figure 3:**
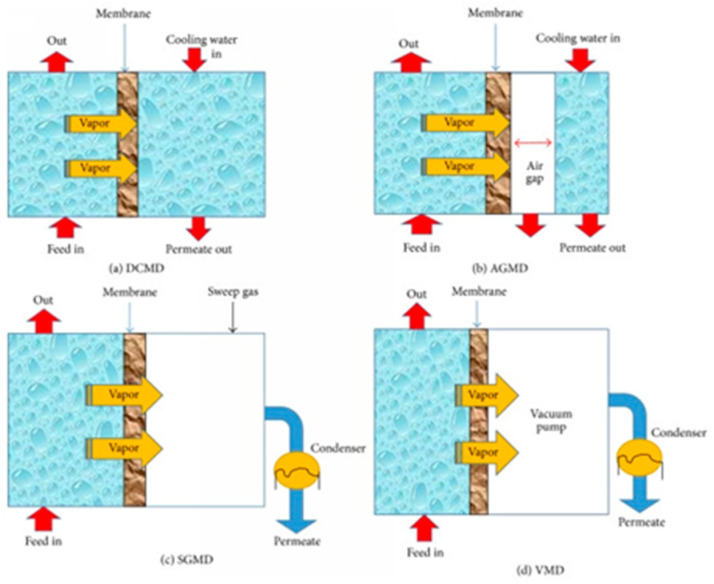
(**a**) Direct contact membrane distillation (DCMD). (**b**) Air gap membrane distillation (AGMD). (**c**) Sweeping gas membrane distillation (SGMD). (**d**) Vacuum membrane distillation (VMD). Reprinted from [38] with open access.

To obtain trans-membrane vapor pressure difference across the two sides of the membrane, membrane distillation can operate in different configurations, of which the most common are as follows: direct contact membrane distillation (DCMD), air gap membrane distillation (AGMD), sweeping gas membrane distillation (SGMD) and vacuum membrane distillation (VMD) [37]. The schematic representation of the different configurations is given in Figure 3.

#### 3.2.1. Direct Contact Membrane Distillation

The simplest membrane distillation setup is direct contact membrane distillation. In this configuration, the membrane is in direct contact with the feed and the permeate. As the feed solution is hot, evaporation takes place at the feed-membrane side. The temperature difference between the two solutions built transmembrane vapor pressure that causes movement of the vapor flux across the membrane toward the permeate side. The vapor condenses in the cold permeate inside the membrane module. The hydrophobic behavior of the membrane prevents the penetration of the liquid feed into the membrane. The main drawback of this distillation setup is the heat loss by conduction during the process [39].

#### 3.2.2. Air Gap Membrane Distillation (AGMD)

In this configuration, the hot feed solution is in contact with the one side of the membrane. Between the condensation surface and membrane, a stagnant air gap is established [40]. The vapors cross this air gap and condense above a cold surface inside the membrane module on the permeate side. Heat loss by conduction is reduced, but resistance to mass transfer is produced with a decrease in flux. This configuration is also utilized for the separation of volatile components from the solution [41].

#### 3.2.3. Sweeping Gas Membrane Distillation (SGMD)

In this membrane distillation assembly, inert gas is used to sweep vapors across the permeate side of the membrane, which condenses outside the membrane module in an external chamber. This is the combination of DCMD and AGMD to reduce heat loss by conduction at reduced mass transfer resistance, respectively [42]. The limitation of this configuration is that a large sweeping volume of gas is utilized for a small volume of the permeate to diffuse [43]. Therefore, the condenser has to do much more work in the SGMD configuration, as a small volume of permeate is vaporized into a large volume of sweep gas.

#### 3.2.4. Vacuum Membrane Distillation (VMD)

In vacuum membrane distillation, the vacuum is generated at the permeate side of the membrane with the help of the vacuum pump. In this case, the condensation takes place outside the membrane module. Heat loss by conduction in this distillation configuration is negligible with high permeation flux. The main disadvantage is the higher probability of membrane wetting [44].

### 3.3. Types of Membrane Geometries

Two membrane geometries are used in the MD: hollow fiber and flat sheet. Although hollow fibers have a large surface-to-volume ratio and large packing density as compared to flat sheet, they offer lower flux owing to poor flow dynamics (usually 1–4 L/m^2^h) in the range of 40–60 °C [45,46] and a high degree of temperature polarization. However, there has been significant progress in the fabrication of hollow fiber membrane designs for MD. This includes hollow fibers having spongy structure with thin walls. Additionally, recent advancements have been made for dual-layer hydrophilic–hydrophobic fibers having a thin efficient hydrophobic (50 µm) PVDF active layer. They have the flux in the range of 50–70 L/m^2^h at about 80–90 °C [47]. The flux reported using flat-sheet configuration is 20–30 L/m^2^h when the inlet temperatures are 60 °C (hot side) and 20 °C (permeate side), respectively [48]. The flux in membrane distillation depends on many operating parameters, including also the length of the membrane. Therefore, it is more pertinent to compare the membrane performance in terms of the mass transfer co-efficient rather than in terms of flux [49].

### 3.4. Membrane Characteristics for MD

A formidable challenge associated with the scale-up of the laboratory MD plant is the fabrication of the membranes with specific properties. The membrane should possess the following features:

1. A porous structure to reduce thermal conductivity and to increase mass diffusion through pores: The pores must be in the range of 0.1 to 0.4 µm and membrane porosity between 40% and 90% [33,50]. Membranes having higher porosity have increased flux and low heat loss by conduction. In spite of the increased permeate flux, the highly porous membrane is more prone to break because of the low mechanical resistance. Porosity of the membrane can be evaluated by the gravimetric method by measuring the weight of the liquid contained in the pores.
  (3) $$\varepsilon =\frac{(W1-W2)/\rho K}{(W1-W2)/\rho k+W2/\rho p}\times 100\%$$
  where W1 accounts for the weight of a wet membrane, W2 for the weight of a dry membrane, ρk is the density of the liquid, and ρp is the density of the polymer.
2. An optimal membrane thickness: Membrane thickness plays an important role in predicting resistance-to-mass transfer. It should be as thin as possible to increase mass transfer and to have high MD permeability. In contrast, to have maximum heat efficiency and to avoid heat loss by the conduction in MD membrane matrix, the membrane thickness should kept as thick as possible [51]. Weimming Ni et al. evaluated the optimum membrane thickness of the PVDF membrane using simulation studies in the range of 10 to 20 µm [52].
3. Mechanical strength and chemical resistance must be high enough to work for a longer period of time: However, limited studies in the literature have focused on the mechanical properties of the membrane for this application. Reported values showed significant variation with the elastic modulus ranging from (34–3491 MPa), tensile strength from (3.4–57.9 MPa), and elongation at break between 41% and 710%. This wide range indicates that no specific mechanical property requirements have been established for the membranes used in membrane distillation. Additionally, the thermal conductivity of the polymer used in the membranes should be low to minimize heat though conduction. Polymeric membranes commonly used in the MD exhibit thermal conductivity values between 0.1 and 0.5 W·m^−1^·K^−1^ [53].
4. High liquid entry pressure (LEP): LEP is the parameter that determines the wetting of the membrane pore. It is the minimum transmembrane pressure that is required by the feed to enter the hydrophobic layer of the membrane. This critical pressure is associated with the interfacial tension, maximum size, the shape of the membrane pore, and the contact angle made by the feed liquid at the pore entrance. To avoid wetting of the membrane, the hydrostatic pressure must be kept lower than LEP. Liquid entry pressure can be evaluated by the Laplace equation.
  (4) $${\mathrm{LEP}}_{\;W}=-\frac{2\beta \gamma l\;cos\theta }{r\;max}$$
  where β is the dimensionless geometric factor that is determined by the pore morphology (β = 1 for the cylindrical pore), γl is the liquid surface tension expressed in Nm^−1^, cos θ is the contact angle made at the solid/liquid surface between feed solution and membrane surface, and r max is the maximum membrane pore size.

### 3.5. Membrane Materials and Fabrication

One of the main problems associated with the MD membrane is pore wetting during the test. The hydrophobic characteristic of the membrane stops the mass transfer in the form of liquid. The vapor/liquid equilibrium is established at the pore interface. Presently, many hydrophobic polymers have been employed for the preparation of porous membranes such as polyvinylidene fluoride (PVDF), polytetrafluoroethylene (PTFE), and polypropylene (PP) [54]. Properties, the final morphology of the membrane, and its preparation are the critical factors, and they greatly depend upon polymer–solvent interaction.

**Figure 4:**
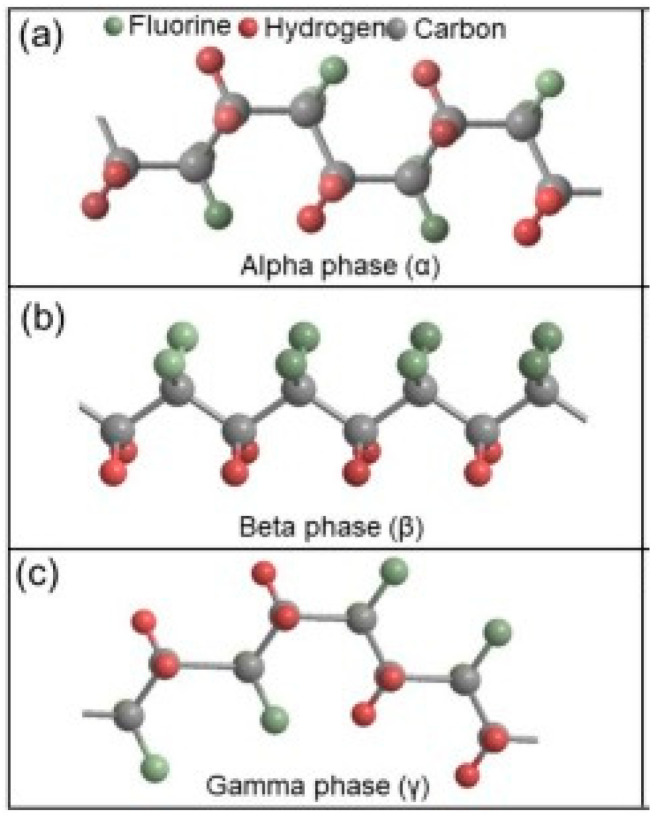
Schematic illustration of the chain arrangement in α alpha (**a**), β beta (**b**), and γ gamma (**c**) phases of PVDF. Reprinted from [59] with open access.

PVDF is the most explored polymer reported in the literature for its usage in UF, MF, fuel cells, and in membrane contactor units for the desalination of brackish and sea water [55]. PVDF gives ease of producibility, and it has been largely explored also in membrane distillation. The vinylidene fluoride monomer polymerizes via free radical polymerization to form PVDF. As a result of different degrees of polymerization, it has different molecular weights. PVDF is a fluoro-polymer, has a semi-crystalline structure, exhibits both amorphous and crystalline phases, and the crystallinity of the polymer ranges between 35 and 70% [56]. Its crystalline phase has three unique molecular conformations and can display five distant crystal polymorphs: α (phase II), β (phase l), γ (phase III), δ, and ε phases based on thermal and mechanical history [57]. Among them, the most common phases are α, β, and γ as indicated in Figure 4 [58,59]. The α phase exists in the monoclinic crystal form and is the most common non-polar phase formed by the (CH_2_–CF_2_)n monomer. In contrast, the β phase of the polymer exhibits polarity and stands as thermodynamically stable configuration. The later phase finds applications in piezo and pyroelectric fields.

PVDF has a mass density and melting point of 1.77 kg/m^3^ and 170 °C, respectively. Its glass transition temperature lies in the range of −40 to −30 °C [58]. It has high chemical inertness, heat resistance, and mechanical strength, which makes it suitable for use in MD. It decomposes at temperatures higher than 316 °C. The physical chemical properties of PVDF polymers are listed in Table 1 below.

PVDF is easily produced and has been extensively explored in membrane distillation. Porous PVDF membranes can be prepared by solvent inversion techniques such as thermal-induced phase inversion (TIPS) [61], non-solvent-induced phase inversion (NIPS) [62], and vapor-induced phase separation techniques (VIPSs). Among all of them, NIPS is the most employed technique for producing membranes for application in membrane distillation. The conventional preparatory method includes dissolving PVDF powder with or without additives in a solvent or in a mixture of solvents with continuous heating and stirring until a homogeneous solution is formed, called dope solution. The prepared solution is then cast on the support (be glass or non-woven polyester) with the help of the casting knife to obtain the flat-sheet configuration. The membrane is then immersed into the non-solvent bath, phase separation occurs, and a membrane is formed. Solvent/non-solvent exchange results in microporous membrane formation.

Currently, the large industrial production of membranes relies on the use of conventional toxic solvents like *N*,*N*-dimethylacetamide (DMAc), 1-methyl-2-pyrrolidinone (NMP), and dimethyl formaldehyde (DMF), etc. These solvents are harmful to human health and environment and face restrictions from the regulatory authorities of the European Union. In addition, the European Union’s REACH (Registration, Evaluation, Authorization and Restrictions of Chemical Substances) has defined these solvents as unsafe substances of high concern (SVHCs) if exposed for a longer period of time [63], and they also have low biodegradability and require extra prevent measures to discard. Replacing traditional toxic solvents with more environmentally friendly solvents while improving the membrane performance is a challenging task. Recent works on the preparation of membranes using PVDF as polymers and greener/sustainable solvents (like dimethyl isorbide DMI [64], ethyl lactate [65], triethyl phosphate TEP [66], and Cyrene^TM^ [67]) have gained much popularity. Table 2 lists the different non-toxic solvents with their physical properties for the preparation of the flat-sheet PVDF membranes. The preparation of flat-sheet PVDF membranes was mainly carried out by the non-solvent and vapor-induced phase separation technique.

As described above, many works can be found in the literature regarding the replacement of traditional solvents with green ones, in the case of PVDF membranes thanks to its ease of processing. F. Russo et al. adapted phase inversion non-solvent-induced phase separation technique to prepare the PVDF membrane in Tamisolve as solvent. Polyethylene glycol (PEG) and PVP were used as a pore forming agent. The effect of different concentrations of polymer (10 wt%, 15 wt% and 18 wt%) was also investigated. All the produced membranes showed the porosity in the range of 71% to 89%, with the contact angle of the top surface ranging from 75° to 91°. The pore size of membranes lies in between ultrafiltration to microfiltration (0.03–0.17 µm). All the prepared membranes were tested for methylene blue dye rejection, membranes with additive PVP (5 wt%), PEG (20 wt%), and a 15 wt% of polymer gives the highest rejection rate 86% of MB [76]. Yu-Xian Lin et al. [77] prepared PVDF flat-sheet membranes for sea water desalination. A composite membrane of polymethyl methacrylate (PMAA)/polyvinylidene fluoride (PVDF) was prepared via non-solvent-induced phase separation technique (NIPS). The results showed that, by increasing the concentration of PMAA from 0 to 14.4 wt%, there was an increase in the hydrophobic character, pore size, and porosity. The membrane with the highest content of PMAA exhibits a stable flux of 18.5 L/m^2^h. Also, T. Marino et al. [66] produced a PVDF membrane using a non-carcinogenic solvent triethyl phosphate (TEP) for the applications in the UF and MF range. Non-solvent-induced together with vapor-induced separation techniques were employed to prepare membranes. The effect of polyethylene glycol (PEG) as an additive on membrane performance was also investigated. The studies show that the low concentration (10 wt%) of PEG results in asymmetric membranes with pore size in range from 0.01 to 0.1 µm. When the concentration of the pore-forming agent PEG increased to 15 and 20%, the membrane structure slowly changes to sponge-like and symmetric. The change led to an increase in the pore size up to 1 µm. Hao-Ren Yang et al. [74] prepared PVDF membranes using TEP as an alternative to toxic carcinogenic solvent using the spray-assisted non-solvent-induced phase separation method (SANIPS). Before the completion of phase inversion, phase water was sprayed on the newly developed membrane and achieved super hydrophobicity with a water contact angle of 154°. The prepared membranes were tested in the direct contact membrane distillation with 10 wt% of NaCl as a feed solution. It showed a stable flux of 22 L/m^2^h, which was much higher than membranes produced through the non-solvent-induced phase separation technique, i.e., 12 L/m^2^h. C. Ursino et al. [61] successfully prepared PVDF flat-sheet membranes by the thermal-induced phase separation technique, using three non-toxic solvents: acetyl tributyl citrate (ATBC), acetyl triethyl citrate (ATEC), and acetyl triethyl citrate (TEC). The pore size of the membranes with a 200 µm knife and blade gap is 0.86 µm for ATBC, 2.44 µm for ATEC, and 3.90 µm for TEC. The pore size increased in correlation with the solubility of the PVDF in the solvent, following the order ATBC < ATEC < TEC. The higher miscibility of PVDF and TEC led to significant solid–liquid (S-L) phase separation, followed by spherulite formation. During the TIPS process, TEC molecules accumulated in solvent-rich regions, which, upon solvent removal, resulted in the formation of larger pores. The observed trend in the membrane pore size is persistent with the solubility profiles of the polymer/solvent. Siamak Nejati et al. [78] fabricated a PVDF membrane with TEP as the solvent via the non-solvent-induced phase separation method. The membranes formed showed asymmetric morphology, characterized with a dense top layer followed by a porous bottom side. The coagulation bath in the case comprises 30 *v*/*v*% 2-propanol in water. The membranes were tested in direct contact membrane distillation with 1 M NaCl as feed solution. The effect on permeate flux and salt rejection with a change in orientation of the membrane in MD was studied. The membrane has greater flux when the top dense side of the membrane faces hot feed solution with a flux value of 26 L/m^2^h; in contrast, a lower flux of 6.2 L/m^2^h was observed when the porous surface is exposed to the hot feed solution. Marino et al. [69] prepared PVDF membranes without using a pore-forming agent in Cyrene^TM^ as a solvent, and bicontinous membranes were formed using NIPS and VIPS-NIPS. All membranes produced showed a hydrophobic nature with a water contact angle greater than 120° and a pore size of 0.55 µm. Physio-chemical properties of Cyrene^TM^ in comparison with other bio-based solvents are listed in Table 3.

The choice of solvent significantly affects the membrane morphology, mechanical properties, and separation performance, presenting both advantages and disadvantages. While green solvents offer a safer and more sustainable alternative to traditional toxic solvents, their properties can influence key membrane characteristics, such as pore structure, mechanical resistance, and hydrophobicity. Some bio-based solvents, such as GVL and Cyrene™, may lead to reduced mechanical resistance due to their ability to plasticize PVDF, which occurs when these solvents interact with polymer chains, reducing intermolecular forces and increasing chain mobility [82]. This effect can enhance membrane flexibility and processability, but it may also compromise mechanical strength and robustness, particularly under high-pressure or high-temperature conditions, such as those encountered in membrane distillation (MD).

Membrane performance is also strongly influenced by solvent properties. TEP and TamiSolve NxG, for instance, have been successfully applied in MD processes. PVDF membranes prepared with TEP have demonstrated high flux and thermal stability, making them promising candidates for MD applications [83]. Similarly, TamiSolve NxG-based membranes have shown fluxes comparable to or even higher than those of traditional NMP-based membranes [84]. However, despite their good performance, the hydrophobicity and wettability of these membranes require careful optimization to prevent pore wetting, which remains a critical challenge in MD applications. In Table 4, a comparison is reported of the main green solvents explored for PVDF membrane preparation, considering their advantages and limitations.

### 3.6. Performance of Membrane Distillation in Desalination

The rejection rate and flux of different distillation assemblies using different molecular weights of PVDF as a polymer are listed in Table 5 below. Table 5 outlines all the important parameters of a distillation setup using PVDF membranes prepared with highly toxic to less toxic, greener solvents. Siamak Nejati et al. [78] fabricated PVDF membranes using TEP as the solvent; the effect of thickness and porosity was studied to estimate the flux value. In this 1 M NaCl solution, at 60 °C, it can be seen that the membrane with less thickness and more porosity gives the flux of 40 L/m^2^h. Surface free energy (SFE) is the critical characteristic of the membranes used in membrane distillation (MD), with lower SFE generally preferred. Lyly L.H. Ting [87] and colleagues investigated the effect of reduced graphene oxide rGO incorporation into the membrane matrix. Their findings showed that both low and high rGO loadings resulted in a reduction in surface free energy as compared to a pristine PVDF membrane. NMP is used as the solvent in the phase inversion technique to fabricate the membrane. Due to the low evaporation rate, NMP facilitated a more controlled pore size distribution during phase inversion. Additionally, its excellent compatibility with rGO ensured uniform dispersion within the membrane matrix. The introduction of graphene further enhanced membrane hydrophobicity. The recorded flux for the membrane with 0.6 M NaCl was 34.6 L/m^2^h. J.A. Prince et al. [88] fabricated PVDF blended with clay nanoparticles, where an increase in the clay particle concentration enhanced membrane hydrophobicity, a key characteristic for effective membrane distillation (MD). The strong interaction between PVDF and clay particles also improved thermal properties of the membrane. The recorded flux for this type of membrane at 0.6 M NaCl feed concentration was 57 L/m^2^h. Similarly, L. Zhao et al. [89] developed an electrospun PVDF-HFP membrane incorporating activated carbon. The recorded flux for this membrane using a 0.6 M NaCl feed solution at 60 °C was 45.6 L/m^2^h. Despite having a larger pore size of 0.7 µm and higher porosity of 90.5% compared to the clay- based membrane, its flux was lower. This can be attributed to the lower feed temperature, which directly influences the driving force in direct contact membrane distillation. Membranes fabricated using the electrospinning technique with a toxic solvent exhibited a higher flux (50 kg/m^2^h [90]) for the 0.6 M NaCl feed solution at a feed temperature of 60 °C, compared to 15 kg/m^2^h [91] for membranes prepared using the phase inversion technique. The superior flux performance of the electrospun membrane is primarily attributed to their inter-connected pore structure, which facilitates efficient vapor transport. Additionally, the large pore size of 1.6 µm in electrospun membranes, compared to the 0.1 µm pore size in non-solvent-induced phase separation membranes (NIPSs), further enhances permeability. Electrospun membranes also demonstrated lower SFE, low thermal conductivity, and enhanced thermal efficiency, all of which contribute to increased flux. Furthermore, the high porosity (87%) of electrospun membranes significantly improves mass transfer in contrast to the 57% porosity observed in phase inversion membranes. In comparison, PVDF membranes prepared via the phase inversion method using a less toxic, biodegradable solvent, TEP, exhibited a slightly higher flux of 16.1 kg/m^2^h [92] compared to 15 kg/m^2^h [91] for membranes fabricated with the toxic DMAc solvent. The high flux of the membrane prepared with the TEP solvent can occur since the membrane has higher porosity (78.1%) as compared to the membrane produced with DMAc with a porosity of 57%. Safa Saidi et al. [93] used Tamisolve, a greener solvent, to prepare the PVDF flat-sheet membrane, with non-solvent-induced phase separation technique. They tested the membrane using 0.6 M NaCl as feed solution and 36 °C as feed temperature. The flux achieved was 2.2 L/m^2^h. This suggests that, while electrospinning offers superior flux performance, phase inversion with environmentally friendly solvents can still achieve competitive results while reducing solvent toxicity.

## 4. Crystallization

The recovery of minerals can be performed using the techniques of crystallization and freezing. Crystallization is a basic separation technique to isolate and purify the chemical substances from aqueous solutions; in it, the precipitation of the solid solute from the liquid medium happens. The solute crystallizes out as a pure crystal phase. In crystallization, the saturation level is controlled by removing solvents, which shifts the solution towards a super-saturated state. The potential difference between this super-saturated solution and the solid crystal phase drives nucleation and crystals formation [95]. Moreover, also reducing temperature induces crystallization.

Crystallization is one of the most efficient and powerful ways to produce crystals from the mother solution. The process is widely used in many areas of research, like in chemical, the pharmaceutical and food industry, etc. Moreover, as it will be described below, membrane crystallization could also be used for the recovery of various salts from wastewaters along with high-quality water [96], for instance, the recovery of NaCl [97], Na_2_SO_4_ [98], Na_2_CO_3_ [99], removing Ca^2+^ and Mg^2+^ from hard water [100] and LiCl [101]. In addition, it does not require additional supporting materials like catalysts, adsorbents, redox reagents, etc. Despite its wide application range, crystallizers have limitations related to the quality of the product and the finite control of supersaturation. Most crystallization units operate in batch and suffer from hydrodynamic and kinetic changes as the plants scale-up. Traditional crystallization units are neither economically nor environmentally viable due to their high energy demands. Typically, the process relies on evaporators or crystallizer units that heat and subsequently cool the solute [95,102]. Therefore, continuous operating units must be used to increase efficiency and to bring down workload. Advancements in understanding the crystallization process will contribute to the developing sensors that can help in monitoring nucleation, crystal growth size, and supersaturation as well as liquid incorporation in crystals and impurities [103]. Incorporating membranes into the crystallization process leverages their intrinsic properties, such as the membrane surface, which supports heterogenous nucleation and enables controlled nucleation and crystal growth kinetics. Membrane distillation crystallization can be conducted using either a temperature or concentration gradient, particularly for highly concentrated solutions near supersaturation.

### 4.1. Membrane Crystallization

Membrane crystallization (MCr) is gaining much interest from researchers to obtain clean water and high-purity crystals. Membrane-assisted crystallization can be categorized based on working principle. Membrane-assisted crystallization membranes based on pressure-driven nanofiltration NF [104] and RO [105] membranes work on the size exclusion principle, where the stream is concentrated, the solvent in liquid phase is removed, and the solute is retained by the membrane before batch cooling crystallization. Crystals, on the other hand, are recovered in a separate tank at a lower temperature.

Another type of membrane-assisted crystallization is antisolvent membrane crystallization (MAAC), primarily used for heat-sensitive pharmaceutical compounds. In this, the membrane acts as a barrier between the two solutions; the antisolvent is miscible with the solvent but does not dissolve the solute. Therefore, in MAAC, the membrane is used to dose the antisolvent in the crystallizing solution, thus generating its supersaturation and inducing crystallization. The activity gradient in the system drives the diffusion of the antisolvent through the membrane, gradually increasing the concentration of the crystalizing solution. This controlled supersaturation promotes heterogeneous nucleation by lowering the energy barriers for crystal formation. S. Chergaoui et al. [106] fabricated PVDF membranes and tested them in a MAAC setup at 23 °C and 1 atm pressure using antisolvent and crystallization solution velocities of 5 and 3.3 × 10^−4^ m/s, respectively. Glycine dissolved in pure water served as a model crystallizing solution, while ethanol was used as the antisolvent. The fabricated membrane achieved a transmembrane antisolvent flux of 0.6 kg/m^−2^s^−1^. The hydrophobic nature of the membrane contributed to higher supersaturation ratios, an increased nucleation rate, and formation of large-size crystals. As a result, 26 µm prism-shaped glycine crystals were successfully obtained with a membrane of 140 µm thickness. Membrane crystallization includes both the membrane distillation and the crystallization processes. During membrane distillation or osmotic membrane distillation, vapor pressure acts as the driving force. It is a hybrid process in which the solution becomes supersaturated to attain separation and solidification of the components. In this process, the membrane acts as the heterogenous nucleation interface, which lowers the potential barrier for reaching the nucleation stage and sets off the nucleation process [107]. Membrane crystallization is the actually extended form of membrane distillation; the concentration of salts and crystallization take place in the same unit [108]. A. Criscuoli and the co-author used MCr for the first time to crystallize sodium chloride [109]. At a feed temperature of 29 °C, the flux was of approximately of 1 × 10^−4^ kgm^2^s^−1^. The MCr feed solution was concentrated above the super-saturation degree by extracting the water content and producing pure water as permeate, while the retentate was circulated to the crystallization chamber for the recovery of the valuable components present in the feed solution in the form of crystals. H.F. Tan. et al. [110] recovered phosphorus using the membrane distillation crystallization process with a PVDF/MFC (micro-fibrillated cellulose) membrane fabricated via a non-solvent-induced phase separation process. The incorporation of MFC as a filler enhanced the solvent/non-solvent exchange rate during phase inversion, leading to larger pores and higher porosity in the membrane. Additionally, the membrane surface was modified with non-fluorinated octadecyltrichlorosilane (OS), which imparted hydrophobicity, resulting in a water contact angle of 154.4°. This modified membrane achieved a stable flux, recovered 18% of phosphate relative to the initial concentration and exhibited a rejection rate greater than 99%. The process produced needle-like struvite crystals with a diameter smaller than 3 µm and length ranging from 4 to 40 µm.

### 4.2. Principles of Membrane Crystallization

Membranes in the MCr play a dual role: they provide the surface for mass transfer and promote crystal nucleation by lowering the energy barrier required to initiate the nucleation. Membranes lower the induction time for the nucleation. This speeds up the process as well as ensures uniform crystal size distribution (CSD) [111]. Membrane crystallization works on two principles: (1) membranes to achieve supersaturation of the solution to initiate nucleation and (2) membranes for the crystal growth process.

#### 4.2.1. Achievement of Supersaturation

In concentrative crystallization, the membrane surface is not used directly for crystal growth but for dehydration of the solution. In this case, crystallization initiates in the bulk when the concentration of feed reaches a certain level. Thus, the solution becomes highly concentrated over time. Pressure-drive membrane processes such as nanofiltration (NF) and reverse osmosis (RO) can be utilized for concentrative crystallization [112]. The key benefit to this process is that, since no membrane is directly involved in the crystallization step, concentration polarization does not occur. Additionally, as the process refers to separation, almost no feed losses occur (i.e., the components present in the feed are retained and separated effectively from the solvent through the membrane).

#### 4.2.2. Membranes for Crystal Growth

In another approach, the membrane surface serves as the site for heterogenous nucleation as well as concentration of the solution to supersaturation level. The driving force behind the process can be either temperature-induced partial vapor pressure or osmotically controlled vapor pressure difference. The membrane surface enhances the nucleation by accelerating system kinetics towards crystallization. This occurs due to a reduction in Gibbs free energy, promoting a shift towards a more stable, lower energy state. According to classical nucleation theory (CNT), two-phase clusters are known for heterogeneous nucleation and metastable state formation during phase transformation [113]. As a result, the metastable region expands, shifting the kinetically controlled region to a thermodynamically controlled region. Often, the growth of the crystals on the membrane follows a diffusion growth model, but this model only refers to the growth in the single phase, rather than crystals growing in clusters [114]. Curcio et al. [115] proved that the membrane influences the energetic barrier to the nucleation process. This showed that different chemical and physical interactions between the hydrophobic membrane and mother liquor have important effects on the thermodynamics of the formation of critical nuclei. The major disadvantage linked to this process is the wetting and the fouling of the membrane surface due to concentration polarization [116].

### 4.3. Configurations and Membranes for MCr Process

MCr can work in all four membrane distillation configurations, which include direct contact, vacuum, air gap, sweep gas, and also in osmotic membrane crystallization. Among them, direct contact is considered the easiest choice for applications in desalination or the concentration of feed solution where water is the major component of permeate. It is simple to operate; the membrane is in direct contact with the feed liquid and cold permeate. The latter also has the task of condensing the vapors from the feed that move through the membrane pores. In vacuum membrane crystallization, the vacuum pump is used to vacuum the water vapors across the membrane surface and condensed in another section [117]. In contrast to other distillation assemblies, V-MCr offers the advantage of maintaining a high vapor pressure gradient between the two membrane surfaces, which increases mass transfer. Quist-Jensen et al. showed [118] that, while direct contact membrane crystallization is ineffective in crystallizing high soluble salts due to the high osmotic pressure of feed concentration, on the contrary, in V-MCr, the application of the vacuum inside the membrane pores maintains the least resistance to transporting vapors, which thus allows achieving the supersaturation needed for crystallization. The main drawbacks of VMCr are the increase in energy consumption and wetting phenomena. When wetting occurs, before using the membrane again, it must be thoroughly dried and cleaned [119].

In osmotic membrane crystallization, an artificially synthesized solution (draw solution) is separated from the feed solution by the semi-permeable membrane. Here, the driving force is linked to the concentration gradient between the feed and the draw solution, which creates a partial pressure difference across membrane surfaces, resulting in evaporation of the desired species. Under the impact of this, the solvent moves from feed to the draw solution side [120,121]. By doing so, the draw solution is diluted on the permeate side, and, consequently, the driving force decreases over time.

Regarding the membranes used in MCr, Table 6 presents different properties, parameters, and performance of PVDF flat-sheet membranes used in the process. In particular, the lowest and highest trans-membrane fluxes registered were of 1.78 L/m^2^h with a 5.3 M NaCl solution, a feed temperature of 34 °C [117], 20 L/m^2^h with a 4.5 M NaCl solution, and a feed temperature of 60 °C, using a commercial PVDF membrane [122]. Z. Cui et al. [117] prepared Poly(vinylidene fluoride-co-hexafluoropropylene) P(VDF-HEF) flat-sheet membranes using dip-coating techniques and used them in direct contact membrane crystallization. The flux recorded for all the three compositions of flat-sheet PVDF membranes with different pore sizes (AD40H_010 0.47 µm, 220 nm, and 450 nm) with a coating of 0.1 wt% of Hyflon AD40H lies in the range between 1.78 and 2.54 L/m^2^h. The higher flux recorded for the membrane with a pore size of 450 nm (AD40H_045) is due to the reduced thickness, large pore size, and higher surface porosity. Surface porosity plays an important role as the effective membrane surface area is responsible for water vaporization. This also contributed to the lower nucleation time of 322 per min. A higher trans-membrane flux of the membrane AD4H_045 leads to an earlier achievement of the degree of supersaturation, thereby promoting nucleation and secondary nucleation. Chan et al. [122] uses a flat-sheet commercial PVDF membrane and tests it with 4.5 M NaCl feed concentrations. The system achieves the record flux of 20 kg/m^2^h under operating conditions of a 60 °C feed inlet temperature and 20 °C permeate temperature. After 5.3 h, the system experiences a sharp decline in the flux that corresponds to the attainment of the supersaturation conditions. This flux reduction was attributed to the membrane fouling and accumulation of the crystals on the membrane surface, ultimately leading to a complete flux drop. The formation of cubic NaCl crystals was observed, indicating that the membrane functioned as the support for heterogenous nucleation. Membranes fabricated using the greener, biodegradable solvent Tamisolve exhibits a lower flux of 1.82 L/m^2^h when tested with a 5.3 M NaCl feed concentration at 40 °C [93] for 7 h. Despite the lower flux, the membrane achieved formation of uniform cubic crystals with a rejection of 99%. The reduced flux compared to other membranes may be attributed due to the smaller pore size of 0.19 microns. Graphene-modified PVDF membranes with varying concentrations (0.5%, 5%, and 10%) were studied. At the feed side, where the NaCl concentration was 5.3 M and the temperature was 36.5 °C, a flux of 8 L/m^2^h was observed for the membrane with 5% graphene loading with the co-efficient of variation (CV) of 26.7%. The incorporation of the graphene into the PVDF matrix enhanced the availability of nucleation sites, thereby accelerating the crystal growth [123]. M. Frappa et al. [124] prepared membranes with different percentages of the 2D (0.5% BT, 7% BT, and 0.5% G) materials. The highest flux (3.9 Lm^2^/h) was recorded for the 7% bismuth telluride loading. All the membranes were tested using 5 M NaCl at the feed temperature of 34 °C. The incorporation of the 2D material into the membrane matrix contributed to the formation of uniform crystals in all cases. The membrane containing 0.5% graphene displayed the lowest CV values (36.7%) among all the composite membranes. The first crystal was detected at 140 min in the case of PVDF/BT (0.5%). Additionally, the interaction of Bi_2_Se_3_ with interface solution facilitated water molecule adsorption, leading to faster supersaturation, reduced nucleation time, and an accelerated crystal growth rate.

## 5. Conclusions

This review highlighted the advancements in PVDF membranes, with a particular focus on sustainable fabrication using greener solvents for membrane distillation and membrane crystallization for NaCl salt solution. PVDF membranes were explored, aimed to address the environmental issue in synthesizing membranes with toxic solvents.

Factors such as surface roughness, charge, pore size, and membrane hydrophobicity were identified as critical parameters influencing performance and mechanical stability. In recent years, research has been conducted in replacing the conventional toxic solvents with alternative solvents of greener characteristics. The membranes should have the capacity to retain hydrophobicity throughout the experiment, have antifouling properties, high mechanical strength, and chemical resistance. In this respect, much research is focused on the tuning of the membrane properties like roughness and charge on membrane surface and pore size. Membrane distillation and membrane crystallization were reviewed as promising thermally driven separation technologies capable of addressing global water scarcity and resource recovery needs. PVDF membranes played a role in these processes to achieve high rejection rates and the effective separation of water and valuable solutes, such as salts. Membrane distillation covers a wide range of applications, including desalination, wastewater treatment, and the recovery of low-boiling-point compounds. It offers advantages in terms of energy efficiency and scalability, while MCr provides an integrated approach to clean water production and crystallization of valuable salts from concentrated solutions. The combination of MD and MCr technologies with PVDF membranes fabricated using greener solvents marks a significant step forward in addressing global water scarcity and promoting sustainable resource management. The major challenge reported, until now in the transition to environmentally friendly alternative greener solvents, is the performance of the membrane and competitive pricing. Membranes fabricated with TEP as a green solvent exhibited a lower flux compared to the commercial membrane prepared with a hazardous solvent [92,94]. Although extensive research has been conducted on membrane distillation using different green solvents, to the best of the authors’ knowledge, only one paper was published for membrane crystallization for sea water using Tamisolve [93] as a non-toxic solvent. The shift away from traditional toxic solvents marked a critical step toward aligning membrane production with environmental sustainability goals. Despite the progress, significant challenges remain in developing membranes that could maintain their performance under harsh conditions, exhibit antifouling properties, and retain hydrophobicity during long-term applications. The need for continued innovation in membrane fabrication methods, such as surface modifications and electrospinning, remain essential to improve pore size control, mechanical strength, and chemical resistance. More efforts are still needed in investigating new greener solvents for membrane preparation to be used in large industrial scale testing for MD and MCr. In fact, the application of these membranes on an industrial level is still hindered by a number of factors like stability of the membrane under harsh conditions and increases in the trans-membrane flux. In the future, the application of greener PVDF membranes in MD and MCr is expected to play a pivotal role in sustainable water treatment. However, large-scale adoption will require addressing several barriers, including improving membrane stability, increasing flux, and ensuring economic viability. Collaborative efforts among researchers, industry, and policymakers will be essential to promote green membrane technologies, reduce carbon footprints, and contribute to circular economy models. Ultimately, fostering innovation in green chemistry and membrane engineering will pave the way for more sustainable and efficient water treatment solutions. Separation and wastewater treatments made through green and sustainable membranes help to reduce the carbon footprints and address climate change, water deficit, aquatic biome and biodiversity. Through Life Cycle Assessments (LCAs), a comparison can be drafted identifying where green solvents can reduce environmental carbon footprints of membranes [126]. However, these membranes should be stable enough to withstand a robust environment. Despite considerable investigations made into the bio-based greener membranes, there still exists a gap in providing the information regarding the scale-up and reuse of these membranes. Addressing these issues is necessary to fully exploit the use of sustainable membranes in the membrane application of water treatment. Regardless of the well-documented harmful effects of the conventional solvents, they continue to be widely used in membrane fabrication due to greater feasibility in productions of large scale and low cost, exhibiting less viscosity and having the ability to solubilize a large number of polymers. Further innovation and research are required to increase the performance and economic viability of the “greener” approach of making them more assertive than their equivalents. The gap signifies the necessity of the framework to be developed between manufacturers, governments, academia, and consumers to contribute to the circular economy for the potential of cost benefits. Actually, green solvents are not economically feasible due to their cost. However, once their use will be adopted in large-scale membrane manufacturing, it is expected that their cost will significantly reduce. Nevertheless, the use of green solvents will decrease the disposal costs of streams containing harmful substances, with consequent benefits for the environment and operators. Moreover, they allow us to avoid specialized control measures needed during the membrane production with traditional solvents. Green membranes support sustainable development goals (SDGs) and the circular economy as they comply with growth principles, in wastewater treatment, energy production, and material separation.

## Figures and Tables

**Table 1:** Physical and chemical properties of PVDF polymers.

Characteristics	Polymer
Structure	(CH_2_-CF_2_)_n_ [60]
Crystalline forms	α, β, γ, δ and ε
Melting point (β)	170 °C
Density	1.77 kg/m^3^
Glass transition temperature	−40 °C to −30 °C
Decomposition temperature	316 °C

**Table 2:** Green solvents for the preparation of PVDF flat-sheet membranes. Adapted from advancements in sustainable PVDF co-polymer membrane preparation using Rhodiasolv^®^ PolarClean as an alternative eco-friendly solvent.

Solvent	Mol. wt g/mol	Boiling Point °C	Preparation Technique	Ref.
ATEC	318.3	132	TIPS	[61]
ATBC	402.5	173		
([BMIM]PF_6_)	284.1	>340	TIPS	[68]
Cyrene^TM^	128.13	226	NIPS	[69]
			VIPS-NIPS	
			NIPS	[70]
			EIPS	
DMI	174.1	93–95	VIPS-NIPS	[64]
DBM	228.2	281	TIPS	[71]
DMSO	78.1	189	NIPS	[72,73]
EL	118.1	154	EIPS-NIPS	[65]
TEP	182.1	215	NIPS	[66]
			SANIPS-NIPS	[74]
TEGDA	234.2	286	TIPS	[61,75]
TEC	276.2	127	TIPS	[61]

Acetyl ethyl citrate: ATEC; acetyl tributyl citrate: ATBC; 1-butyl-3-methylimidazolium hexafluorophosphate: ([BMIM]PF_6_); dihydrolevoglucosenone: Cyrene^TM^; dimethyl isosorbide: DMI; maleic acid dibutyl ester; DBM; dimethyl sulfoxide: DMSO; ethyl lactate: EL; triethyl phosphate: TEP; triethylene glycol triacetate: TEGDA; and triethyl citrate: TEC.

**Table 3:** Comparison of physical-chemical properties of bio-based solvent Cyrene^TM^, DMI and γ-valerolactone.

Properties	Cyrene^TM^ [69]	Dimethyl Isorbide (DMI) [79]	γ-Valerolactone (GVL) [80]
Color	Colorless Light yellow	Colorless to slight yellow	Colorless
Molecular weight (g/mol)	128.1	174.1	100.1
Formula	C_6_H_8_O_3_	C_8_H_14_O_4_	C_5_H_8_O_2_
Density (g/cm^3^)	1.2	1.1	1.0
Miscibility with H_2_O	Complete	Complete	Complete
PVDF-solvent distance ^a^	3.3	5.7	3.1
δd (MPa ^0.5^) [64]	18.8	17.6	17.1
δp (MPa ^0.5^) [64]	10.6	7.1	11.9
δ_H_ (MPa ^0.5^) [64]	6.9	7.5	6.2

^a^ Distance between the PVDF solvent is calculated using the following equation reported in [81].

**Table 4:** Comparison of green solvents used for PVDF membrane preparation as alternatives to traditional toxic solvents (NMP, DMF, DMAc). Adapted from [82,85,86].

Green Solvent	Advantages	Disadvantages
TamiSolve NxG (*N*-butyl pyrrolidone)	- Non-toxic and biodegradable solvent. - High solubility for PVDF, enabling membranes with a well-controlled structure. - Completely water-miscible, allowing easy removal after fabrication.	- High boiling point (241 °C) requires optimized drying conditions to prevent defects at industrial level.
Dimethyl Sulfoxide (DMSO)	- Less toxic alternative to NMP and DMF. - Excellent solubility for PVDF, allowing controlled membrane morphology. - Widely available at low cost.	- Low volatility requires longer drying times to avoid structural defects. - Residual traces may affect membrane properties.
Triethyl Phosphate (TEP)	- Good compatibility with PVDF. - Produces membranes with controlled porosity and reduced macrovoid formation. - High thermal stability and well suited for large-scale applications.	- Less commonly used in industrial-scale membrane production.
Dimethyl Isosorbide (DMI)	- Sugar-based solvent with strong PVDF solubility. - Good water miscibility, facilitating easier post-processing. - Suitable for membrane fabrication via VIPS and NIPS.	- Limited industrial adoption compared to traditional solvents. - Higher cost than petroleum-based alternatives.
γ-Valerolactone (GVL)	- Sugar-based solvent with strong PVDF compatibility. - Excellent dissolution properties without requiring toxic co-solvents. - Readily biodegradable.	- May lead to reduced mechanical strength in membranes due to polymer plasticization. - Can affect membrane robustness in pressure-driven applications.
Dihydrolevoglucosenone (Cyrene™)	- Sugar-based solvent derived from biomass, fully sustainable. - Enables the formation of membranes with high porosity and well-controlled structures. - Non-toxic and biodegradable.	- High viscosity can complicate membrane fabrication. - Can plasticize PVDF, reducing mechanical strength under operational stress.
PolarClean (Methyl 5-dimethylamino-2-methyl-5-oxopentanoate)	- Derived from industrial waste valorization (highly sustainable). - Produces membranes with high permeability and stable mechanical properties. - Water-miscible, facilitating post-processing cleaning.	- High viscosity can affect processability. - Limited commercial availability and higher cost compared to traditional solvents.

**Table 5:** Properties, parameters, and performance of the flat-sheet PVDF membranes prepared by using toxic and greener solvents in direct contact membrane distillation.

	Dope Solution Preparation		Membrane Preparation	Membrane Characterizations				DCMD Condition		Membrane Performance		Ref.
	Additives	Solvent	Method	Contact Angle °	Pore Size µm	Porosity %	Thickness µm	Temp °C	Feed Conc. M NaCl	Flux kg/m^2^h	Rejection	
PVDF	-	TEP	NIPS	125 ± 2	-	70	60	60	1	32	-	[78]
PVDF	-	TEP	NIPS	125 ± 2	-	70	150	60	1	20	-	[78]
PVDF	-	TEP	NIPS	125 ± 2	-	75	60	60	1	40	-	[78]
PVDF	-	TEP	NIPS	125 ± 2	-	75	150	60	1	26	-	[78]
PVDF	rGO	NMP	NIPS	150.7 ± 2.8	-	-	-	60	0.6	34.6	99.9	[87]
PVDF	TiO_2_ and FTCS (for dip coating)		Dip coating on commerical PVDF membrane	166	0.4	-	-	70	0.6	75	94	[94]
PVDF Kynar^®^	Cloisite^®^	DMAc and acetone	Electrospinning	154.2 ± 3.0	0.6 ± 0.2	81 ± 3	-	65	0.6	57	99	[88]
PVDF Kynar^®^	Fe_3_SO_4_	DMAc	NIPS	99.2	0.1	57	-	60	0.6	15	99.5	[91]
PVDF Kynar^®^	-	TEP	SANIPS	154	-	93	83	60	1.8	22	>99.9	[74]
PVDF-HFP	AC	DMF (0.005 wt% LiCl)	Electrospun	142.7 ± 0.6	0.7 ± 0.036	90.5 ± 1.7	120	60	0.6	45.6	99.9	[89]
PVDF-HFP		TEP	NIPS	89.2	0.06	78.1	49 ± 8	60	0.6	16.1	99	[92]
PVDF-HFP	PEG	Tamisolve	NIPS	89.1 ± 0.1	0.05 ± 0.01	77.2 ± 0.8	148.7 ± 2.2	36	0.6	2.2	99.9	[93]
PVDF/TBAHP/PS; 35 wt%		DMF and acetone	Electrospinning	151.7	1.6	87	100 ± 10	60	0.6	50	99.9	[90]

Phyllosilicate: Cloisite^®^; dimethylacetamide: DMAc; activated carbon: AC; tetrabutylammonium hexafluorophosphate: TBAHP; polystyrene: PS; dimethylformamide: DMF; lithium chloride: LiCl; fluorosilanization: FCTS; titanium dioxide: TiO_2_; reduced graphene oxide: rGO; ferric sulfate: Fe_3_SO_4_; *N*-Methyl-2-pyrrolidone: NMP; polymethyl methacrylate: PMMA; polyethylene glycol: PEG; and triethyl phosphate: TEP.

**Table 6:** Properties, parameters, and performance of the flat-sheet PVDF membranes prepared by using toxic and greener solvents in membrane crystallization.

Type of PVDF	Dope Solution Preparation		Membrane Preparation	Membrane Characterization				MCr/MDC Conditions				Membrane Performance	Ref.
	Additives	Solvent	Preparation Method	Contact Angle °	Pore Size µm	Porosity %	Thickness µm	Application	Feed Temp. °C	Permeate Temp.°C	Feed Conc. M NaCl	Flux kg/m^2^h	
PVDF- VVHP04700	Hyflon AD40H (for dip coating)	-	Dip coating	132 ± 1	0.4	59.4 ± 0.8	102.9 ± 2	MCr	34	10.5	5.3	1.7	[117]
PVDF- HVHP04700	Hyflon AD40H (for dip coating)	-	Dip coating	137 ± 1	0.8	58.3 ± 0.5	98 ± 2.5	MCr	34	10.5	5.3	2.5	[117]
PVDF	LiCl	DMF	Wet phase inversion	98.5	0.3	44.9	66	MDC	69.8	26.8	5.1	1.4	[125]
PVDF	Acetone	DMF	Wet phase inversion	80	0.32	41.1	60	MDC	69.8	26.8	5.1	1.7	[125]
PVDF	-	-	Commercial membrane	-	0.2	70	125	MCr	60	20	4.5	20	[122]
PVDF	Graphene pallet	NMP	Dry–wet phase inversion	156 ± 5	0.7	86	-	MCr	36.5	-	5.3	8	[123]
PVDF/BT (0.5%)		NMP ink	Dry–wet phase inversion	128 ± 8	0.5 ± 0.2	75 ± 1	68 ± 1	MCr	34	11	5	2.7	[124]
PVDF/BT (7%)		NMP ink	Dry–wet phase inversion	130 ± 2	0.5 ± 0.08	77 ± 1	100 ± 5	MCr	34	11	5	3.9	[124]
PVDF/G (0.5%)		NMP ink	Dry–wet phase inversion	136 ± 1	0.2 ± 0.05	56 ± 7	62 ± 3	MCr	34	11	5	1.6	[124]
PVDF-HFP	PEG	Tamisolve	NIPS	88.02 ± 0.6	0.19 ± 0.03	80.1 ± 1.3	142.2 ± 4.2	MCr	40	10	5.3	1.8	[93]

Lithium chloride: LiCl; dimethylformamide: DMF; *N*-Methyl-2-pyrrolidone: NMP; bismuth telluride: BT (Bi_2_Te_3_); and polyethylene glycol: PEG.

## Data Availability

The raw data supporting the conclusions of this article will be made available by the authors on request.
